# Discrimination of CRISPR/Cas9-induced mutants of rice seeds using near-infrared hyperspectral imaging

**DOI:** 10.1038/s41598-017-16254-z

**Published:** 2017-11-21

**Authors:** Xuping Feng, Cheng Peng, Yue Chen, Xiaodan Liu, Xujun Feng, Yong He

**Affiliations:** 1 Key Laboratory of Spectroscopy, Ministry of Agriculture, College of Biosystems Engineering and Food Science, Zhejiang University, Hangzhou, 310058 China; 2 Institute of Quality and Standard for Agro-products, Zhejiang Academy of Agricultural Sciences, Hangzhou, 310021 China; 3 Institute of horticulture, Zhejiang academy of agricultural science, Hangzhou, 310021 China

## Abstract

Identifying individuals with target mutant phenotypes is a significant procedure in mutant exploitation for implementing genome editing technology in a crop breeding programme. In the present study, a rapid and non-invasive method was proposed to identify CRISPR/Cas9-induced rice mutants from their acceptor lines (huaidao-1 and nanjing46) using hyperspectral imaging in the near-infrared (NIR) range (874.41–1733.91 nm) combined with chemometric analysis. The hyperspectral imaging data were analysed using principal component analysis (PCA) for exploratory purposes, and a support vector machine (SVM) and an extreme learning machine (ELM) were applied to build discrimination models for classification. Meanwhile, PCA loadings and a successive projections algorithm (SPA) were used for extracting optimal spectral wavelengths. The SVM-SPA model achieved best performance, with classification accuracies of 93% and 92.75% being observed for calibration and prediction sets for huaidao-1 and 91.25% and 89.50% for nanjing46, respectively. Furthermore, the classification of mutant seeds was visualized on prediction maps by predicting the features of each pixel on individual hyperspectral images based on the SPA-SVM model. The above results indicated that NIR hyperspectral imaging together with chemometric data analysis could be a reliable tool for identifying CRISPR/Cas9-induced rice mutants, which would help to accelerate selection and crop breeding processes.

## Introduction

Crop breeding has come to the stages of targeted genome editing that has benefited from the development of the CRISPR/Cas9 (clustered regularly interspaced short palindromic repeats/CRISPR-associated nuclease 9, Cas9) gene editing system. CRISPR/Cas9-mediated targeted gene modification was first introduced for plant genome editing and crop breeding purposes in 2013^1^. This revolutionary technology allows plant breeders to target very specific pieces of DNA without introduction of foreign DNA and to influence key traits accurately and quickly^2^. Although the CRISPR/Cas9 system is an excellent tool for genome editing, the next major challenge is to identify the resulting mutants due to the extent of off-target mutations and the differences in cleavage specificity among species^3^. Several approaches have been developed for identifying the mutants produced by the CRISPR/Cas9 system, such as T7 endonuclease I (T7EI) assay, high-resolution melting curve analysis (HRAM), restriction fragment length polymorphism (RFLP) and polymerase chain reaction (PCR)/restriction enzyme (RE) assay^4–8^. However, these DNA- and protein-based techniques need the complex process of extractions are time- and labour-intensive, costly, and require expensive capital equipment. Moreover, some precious samples will be destroyed by these techniques.

Therefore, a method for the selection of mutants introduced by the CRISPR/Cas9 system without requiring any wet chemistry, particularly extraction protocols, would be advantageous where large numbers of samples must be analysed. In contrast to biochemical assays, near-infrared (NIR) spectroscopy does not require technical expertise or complex techniques, and the spectrophotometer can be installed anywhere with no requirements for reagents or complicated protocols^9^. The radiation from light sources in the NIR region (750–2500 nm) provides a unique spectral signature in the form of a spectrum corresponding to the energy absorption and consequent vibrations of organic molecules based on relative overtones and combinations of O-H, N-H, and C-H functional groups^10^. Previous research in the transgenic field on the application of NIR spectroscopy and chemometrics has demonstrated this technique’s capacity to identify mutants mediated by transgenic technology^11^. García-Molina *et al*.^9^ applied the NIR spectroscopy combined with partial least square (PLS) analysis to discriminate low gliadin wheat grain from non-transgenic wheat lines with excellent classification accuracy (as high as 96%). Luna *et al*.^12^ used NIR and support vector machine-discriminant analysis (SVM-DA) to discriminate completely transgenic soybean oils from non-transgenic ones. Additionally, NIRs combined with chemometrics have also been employed successfully in the identification of transgenic rice^13^, corn^14^ or mutant of barley^15^. The basis of this technology for the detection of mutants generated using transgenic technology is that it could be used to identify phenotypic changes caused by genotypic changes that ultimately bring about changes in organic molecular bonds^11^. However, until now, no report in the literature has described the application of NIR hyperspectral imaging in CRISPR/Cas9-induced mutant recognition in the efficiency of the plant breeding process. In contrast to conventional NIR spectroscopy, NIR hyperspectral imaging has been used to combine traditional optical imaging and a spectral method and is capable of capturing images over broad contiguous wavelengths in the NIR region and has received much attention in cereal science^16–18^.

Therefore, the main objectives of this study were (1) to study the feasibility of screening CRISPR/Cas9-induced mutant rice seeds using NIR hyperspectral imaging and chemometrics analysis; (2) to identify important wavelengths that can be attributed to the differences between wild-type (WT) and mutant rice strains; (3) to build an optimal discrimination model based on important wavelengths to simplify the prediction model and to speed up the operation; and (4) to visualize the number and locations of mutants by developing imaging processing algorithms.

## Results and Discussion

### The Morphological characterization and thousand-grain weight

According to Ishimaru *et al*.^19^, a deletion *TGW6* mutant had greater grain length and higher thousand-grain weight (TGW) but did not influence grain width compared with its transformation receptors. Table 1 shows the mean results of morphological features and TGW. The mean values for area, perimeter, length in pixels and TGW were significantly different between mutants and the WT at the 0.05 level when using Duncan’s multiple range test. Area, perimeter and length in pixels of seeds were increased after the *TGW6* editing for both huaidao-1 and Nanjing 46. No significant difference of width in pixels was observed between the mutants and the WT (P < 0.05). Increases in TGW of 5.8% and 2.3% were detected for mutants from huaidao-1 and nanjing46, respectively. The bright appearance of seeds is a stable genetic characteristic and was used as one of the assessments for classification. Grain phenotypic changes after gene editing could be acquired by calculating the morphological features from the hyperspectral image of each rice seed in this study. However, the phenotypic changes were slight, and it was difficult to identify mutants by appearances from the images alone.

**Table 1:** Means of shape features and thousand-grain weight (TGW) from the studied rice seeds.

Rice varieties	Area in pixels	Perimeter in pixels	length in pixels		Width in pixels	Thousand-grain weight (TGW)
WT of huaidao-1	61.31 ± 6.32^b^	29.43 ± 1.53^b^	11.20 ± 0.61^b^		5.65 ± 0.44	24.56 ± 0.64^b^
Mutants of huaidao-1	65.28 ± 6.29^a^	30.95 ± 1.65^a^	11.76 ± 0.62^a^		5.73 ± 0.37	25.99 ± 0.61^a^
WT of nanjing46	54.73 ± 5.90^b^	27.96 ± 1.62^b^	10.34 ± 0.61^b^		5.33 ± 0.45	25.66 ± 0.54^b^
Mutants of nanjing46	55.88 ± 7.99^a^	28.18 ± 2.47^a^	10.85 ± 0.72^a^		5.36 ± 0.41	26.25 ± 0.30^a^

Different letters denote significant differences between wild-type (WT) and CRISPR/Cas9-induced mutants (small letters) by Duncan’s multiple range test (P < 0.05). Values are presented as the means ± SD.

### Overview of spectra

**Figure 1:**
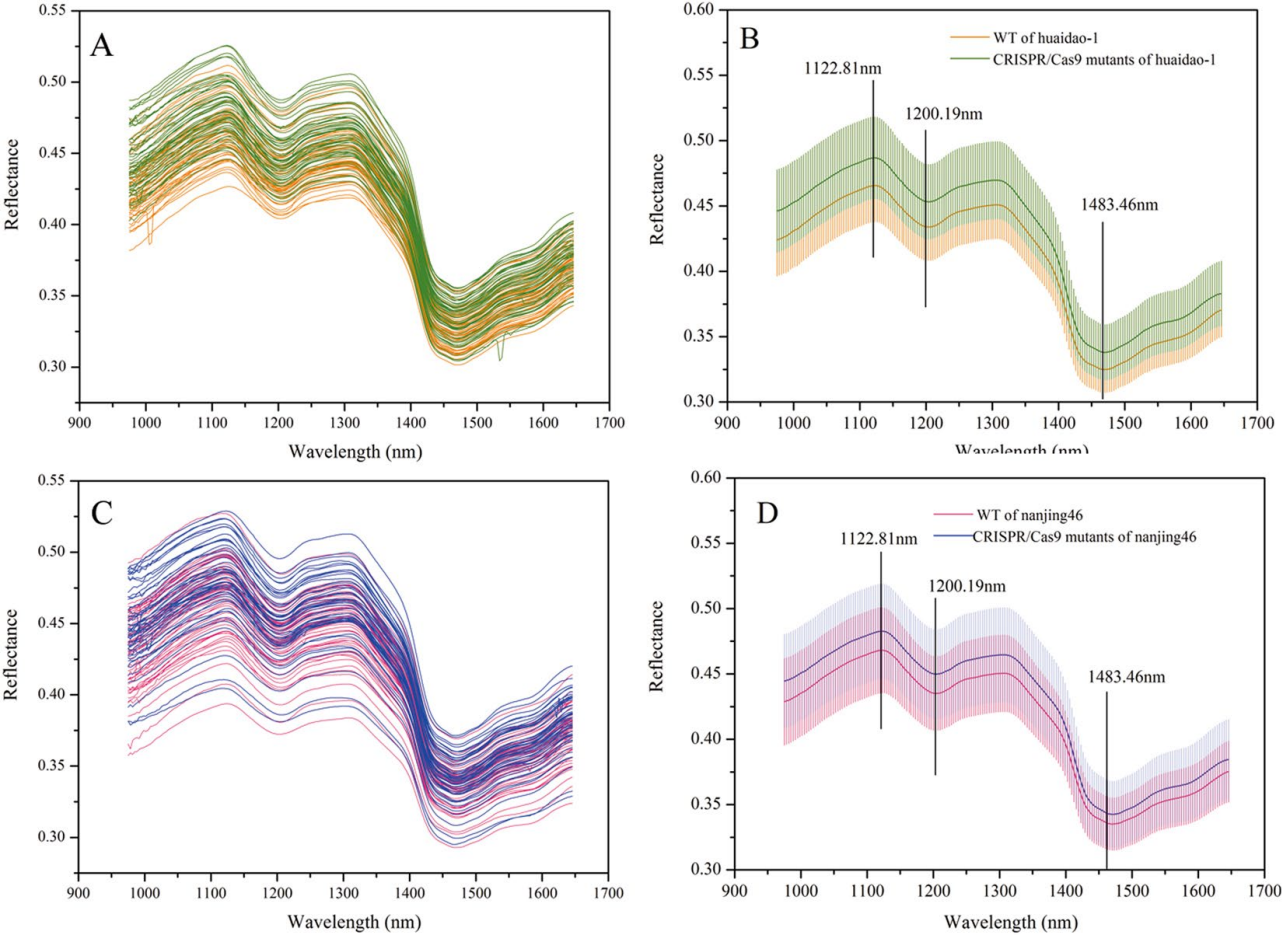
Profiles for raw spectra (**A**,**C**) and average spectra (**B**,**D**) of wild-type (WT) and CRISPR/Cas9-induced mutants from huaidao-1 (**A**,**B**) and nanjing46 (**C**,**D**) rice seeds. The shaded area represents the standard deviation in each wavelength.

Only the spectrum range of 975–1646 nm was used for analysis due to the back-end noise caused by the detection instrument. Figure 1(A,C) shows the extracted spectra of the ROI, and Fig. 1(B,D) shows the mean and standard deviation spectra of the WT and mutant samples. The patterns of the reflectance curves were similar to those of other crop seeds, such grape seeds^20^, oat and groat samples^21^, and *Jatropha curcas* L seeds^22^, although the position and magnitude of the valleys were specific. The NIR spectra information mainly described the shell chemical compositions as the rice seeds were unshelled. There was a peak at approximately 1122.81 nm and valleys at approximately 1200.19 and 1483.46 nm. Two absorption bands at approximately 1122 and 1200 nm contributed to the second overtone of C-H stretching vibrations of carbohydrates^21,23^, and the band at 1483 nm was related to the N-H stretch first overtone from CONH_2_^24^. The graphic also shows that the curves of the spectra from the different genotypes show consistent trends, and they have similar peaks and valleys positions. Slight differences could were noted from the average spectra of WT and CRISPR/Cas9-induced mutant rice seeds of these two varieties. The mutant rice seeds exhibited higher spectral reflectances throughout the entire NIR spectrum than WT for both huaidao-1 and nanjing46. Therefore, the differences in the extents of magnitude of NIR reflectance might result from the phonotypic changes that result from the CRISPR/Cas9-medited genome editing.

### Classification analysis by PCA

**Figure 2:**
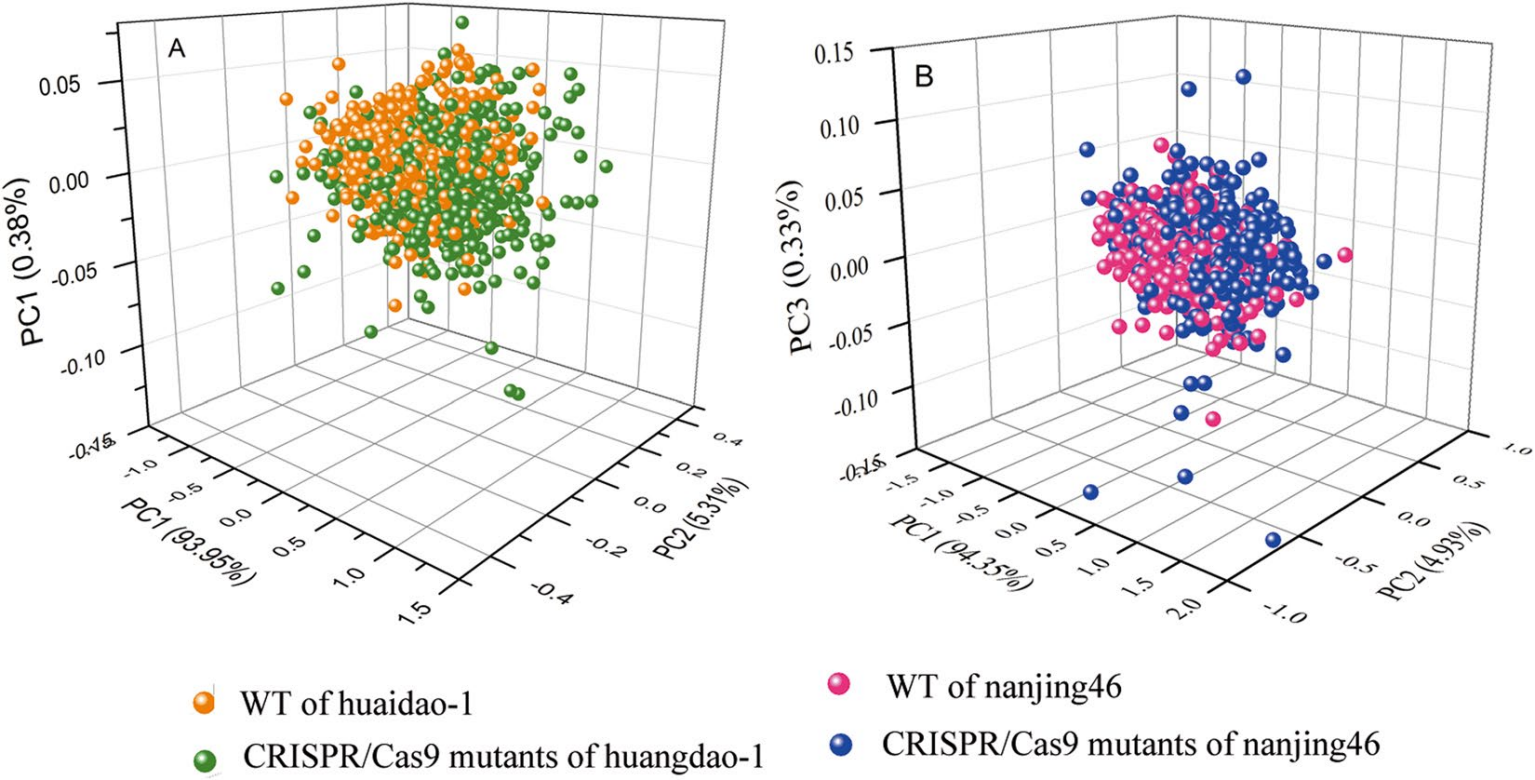
Three-dimensional (3D) principal component score plots of the first three PCs based on the average spectrum of each sample. (**A**: huaidao-1; **B**: nanjing46).

PCA is an effective algorithm for reducing the dimensionality of data into a set of principal components (PCs), solving the problem of multicollinearity and handling potential co-linearity between variables. In this case, PCA programmes developed on all of the average wavelengths of rice samples were first applied to visualize the possible clusters and trends in a PCA score plot. The three-dimensional (3D) principal component score plot of the first three PCs of rice samples is illustrated in Fig. 2. The first three PCs explained the most spectral variations at 99.64% (93.95%, 5.31%, 0.38% for PC1, PC2, PC3, respectively) for huaidao-1 and 98.61% (94.35%, 4.93%, 0.33% for PC1, PC2, PC3, respectively) for nanjing46. As can been seen from Fig. 2, mutants were clustered together from their acceptor lines, but there were still several overlapping places in the PCA score plot. The discrimination between WT and mutants was not clear for huaidao-1 and nanjing46. It is worth mentioning that the target gene edited by the CRISPR/Cas9 system, *TGW6*, resulted in a slight phenotypic change (TGW and enhanced grain length) without a change in grain quality^19^. As consequence, the discriminant analysis established by the PCA was not sufficiently effective to select the CRISPR/Cas9-induced mutants. As none of the PCs alone contained sufficient information to fully segregate whole rice seeds from their acceptor lines, SVM and ELM discrimination models were run in the following study.

### Selection of optimal wavelengths

Hyperspectral imaging data contain redundant information, which affects the prediction performance of the model. Variable selection was performed using PCA-loadings and SPA wavelength selection-based techniques to facilitate and speed up the classification. Table 2 shows the effective wavelengths that were identified using the SPA and PCA-loadings method. By applying the optimal wavelength selection method, these two rice varieties showed mainly similar effective wavelengths at 1122.81, 1200.19, 1227.12, 1314.72, 1402.42, and 1581.51 nm. It is said that NIR spectroscopy is susceptible to the chemical bonding of organic matter molecules, such as C-N, C-H and N-H stretching vibrations, which could be caused by genotypic changes that result in phenotypic changes^11^. Absorption bands at approximately 1122, 1200 and 1314 nm were related to the second overtone of C-H stretching vibrations^21,25–27^. The wavelength of 1402 nm presents the O-H stretch from carboxyl acids^28^. A peak in the vicinity of 1580 nm was determined to be associated with the first overtone of O-H stretching vibrations^29^. These wavelengths carrying the discriminant information are believed to correspond to the NIR spectral bands relevant to changes in a mutant’s properties caused by *TGW6* gene editing by the CRISPR/Cas9 system.

**Table 2:** Sensitive wavelengths selected by SPA and PCA-loadings.

Method	Rice varieties	Number	Optimal wavelengths/nm
SPA	Huaidao-1	12	1011.93; 122.81; 1227.12; 1314.72; 1345.06; 1402.42; 1439.55; 1483.46; 1581.51; 1611.96; 1642.43; 1645.82
	Nanjing46	11	1122.81; 1200.19; 1227.12; 1314.72; 1345.06; 1372.05; 1405.79; 1439.55; 1507.12; 1581.51; 1645.82
PCA-loadings	Huaidao-1	8	1122.81; 1200.19; 1227.12; 1247.33; 1314.72; 1402.42; 1459.81; 1581.51;
	Nanjing46	8	1122.81; 1200.19; 1227.12; 1247.33; 1314.72; 1402.42; 1459.81; 1581.51

### Classification analysis using a discrimination model

PCA is a powerful method for finding the possible clusters of trends among samples; nevertheless, it cannot be applied for building predictive models for classification. Therefore, discriminant models built on both full and effective spectra were proposed to highlight the chemical differences between samples in the latter step (Table 3). The accuracies (in percentages) obtained for the calibration and prediction sets were summarized. The SVM and ELM models all achieved good recognition results in cases of large samples. For samples from Huaidao-1, better discrimination models using full NIR spectra were obtained from SVM, showing excellent accuracies of 92.38% on the calibration set and 92.50% on the prediction set. The classification capacity of the SVM model was also acceptable for Nanjing46, with an accuracy close to 90%.

**Table 3:** Discrimination results of different models for differentiating WT and CRISPR/Cas9-induced rice mutants based on full wavelengths and optimal wavelengths.

Rice varieties	Discrimination model	Model build on full wavelength			Model build on optimal wavelength selected by SPA			Model build on optimal wavelength selected by PCA-loadings		
		Parameter^a^	Calibration set	Prediction set	Parameter	Calibration set	Prediction set	Parameter	Calibration set	Prediction set
Huaidao-1	SVM	256, 3.0314	92.38%	92.5%	256, 48.5029	93%	92.75%	256, 84.4485	86.13%	81%
	ELM	28	90.25%	93.25%	9	91.37%	92%	64	83.38%	81.25%
Nanjing46	SVM	256, 3.0314	89.75%	88%	256, 84.4485	91.25%	89.50%	256, 27.8576	80.5%%	76.50%
	ELM	35	88.62%	90.25%	14	88.75%	90%	35	80.13%	80.75%

^a^par indicates the parameters of the discrimination models, (c,g) for SVM, the optimum number of hidden nodes for ELM.

Normally, hyperspectral images contain high-dimensional data with redundancy among contiguous wavelengths. According to Wold *et al*. (1996) discriminant models based on optimal wavelengths might be equally or more efficient than full spectra^30^. Judicious selection of spectra could discard uninformative wavelengths and decrease sensitivity to non-linearity, which would improve a model’s robustness and improve the model’s operating speed. As can been seen from Table 3, optimal wavelength algorithms affected the performance of mutant detection for both of these rice varieties. The SPA optimal wavelength algorithm improved the performance of the two models, but the PCA loading lines algorithm reduced the models’ recognition capacities. The numbers of effective wavelengths decreased to only 6% for nanjing46 and 5.5% for huandao-1 after using SPA method. The SPA-SVM was suitable for CRISPR/Cas9-induced mutant identification from both rice varieties, as good results were obtained in both the calibration and predication sets. For samples from huaidao-1, correct classifications of more than 90% were obtained in the calculation and predication sets, indicating that these selected spectra had reliable discrimination power for classification. SPA-ELM was only slightly worse than the SPA-SVM model for huaidao-1. For samples from nanjing46, SPA-SVM also improved the classification capacity. The discrimination capacity of the SPA-ELM model was slightly worse than that obtained from all of the wavelengths but was still rated as acceptable. Nearly all discrimination models built on optimal wavelengths selected by the PCA loading lines method yielded worse results than models based on full wavelengths. The overall results indicated that it was feasible to discriminate CRISPR/Cas9-induced mutant seeds using hyperspectral imaging and that the SPA-SVM recognition model was a reliable and robust model. Therefore, only the SPA-SVM discrimination model was used in the further study.

### CRISPR/Cas9-induced mutant visualization

**Figure 3:**
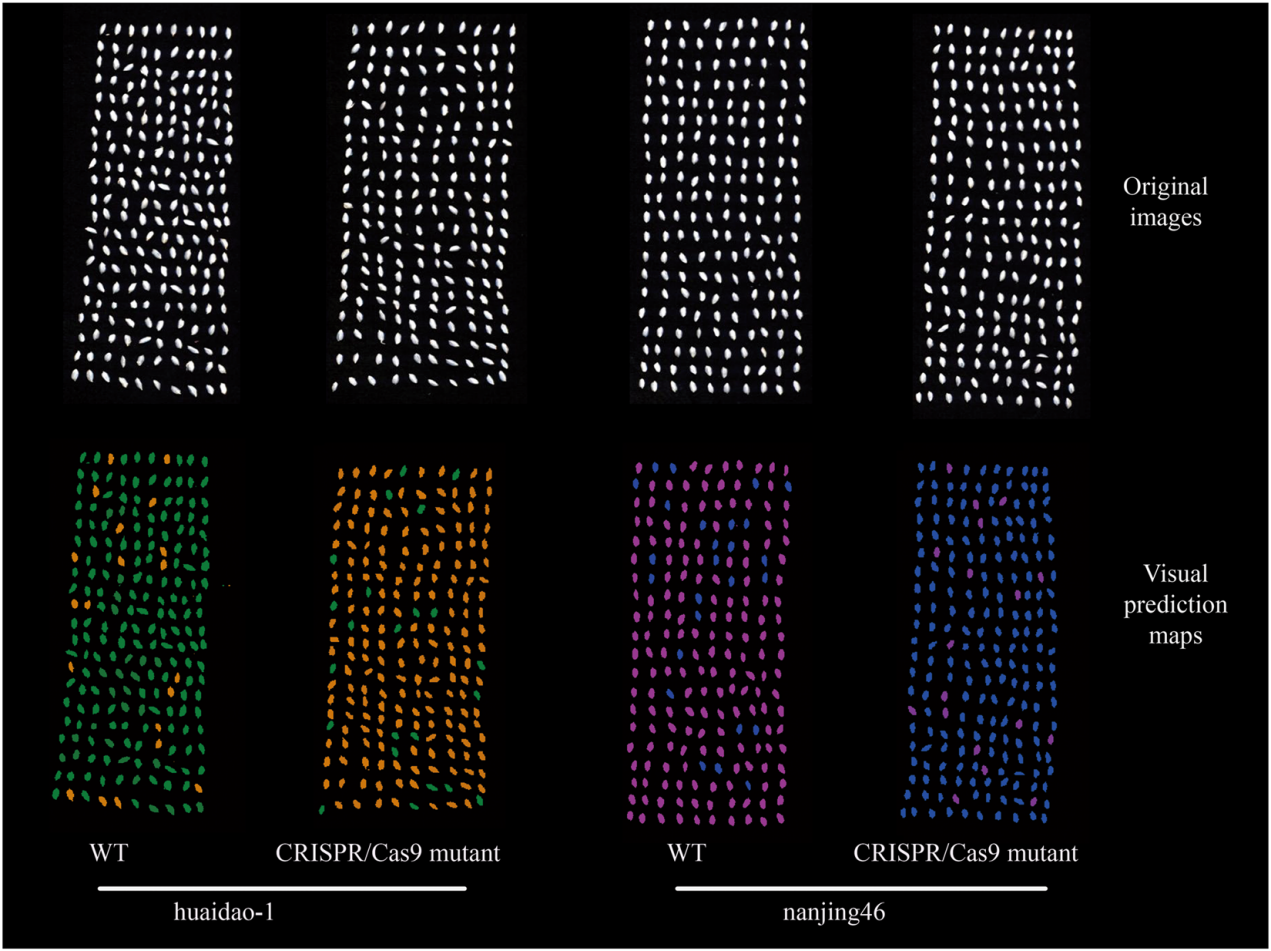
Visual prediction map based on the SPA-SVM model as predictors. The colours (olive: huaidao-1 WT rice seeds; orange: huaidao-1 CRISPR/Cas9-induced mutant rice seeds; pink: nanjing46 WT rice seeds; navy; nanjing46 CRISPR/Cas9-induced mutant rice seeds) correspond to objects identified as class members.

NIR hyperspectral imaging, known as a chemical imaging system, can collect both spatial and spectral information related to chemical constituents of a sample. As described in the Materials and Methods section, discrimination models on the full spectra of all pixels lead to heavy computational burdens and require high-profile computer hardware. In such cases, SVM models developed on optimal wavelengths selected by SPA were applied to the objects’ pixels to predict classes from different genotypes of rice. A very uniform set of seeds (size and morphology) were inspected in this step. The mean pixels of each object were calculated using the object-wise approach from the outset^31^. Figure 3 shows some example of classification maps for identifying mutants. It is impossible to distinguish WT and CRISPR/Cas9-induced mutant seeds from both genotypes of rice by the naked eye; the differences within the same sample could be easily discerned from the final visualization distribution maps. It was noted that some seeds on the classification map were fallible. For samples from huidao-1, the misclassified number were 20 (90.50% accurate classification) for WT and 23 (89.04%) for mutant, respectively. And the predictive accuracies of nanjing46 were 87% and 91.42% for WT and mutant, respectively. That is to say, the SPA-SVM model produced a satisfied classification. The morphological characters of seeds in classification map had some changes due to the low resolution of the NIR imaging system and the image segmentation algorithm. However, the main shapes of the seeds and their locations were clearly shown on the prediction map. The results indicated that NIR hyperspectral imaging together with chemometric analysis was a promising technique to identify and locate single CRISPR/Cas9-induced mutant rice seeds, which has the potential to be a powerful tool for evaluating large numbers of samples from CRISPR/Cas9 gene editing performance trials for breeding programmes.

## Conclusions

CRISPR/Cas9 genome editing technology has shown great potential for targeting gene editing and facilitating crop breeding programmes. However, a time-consuming screening of large populations is required to identify mutants because of extensive potential off-target effects and the targeting specificity of the technology. This paper focused on the use of NIR hyperspectral imaging (975–1646 nm) combined with discrimination model (SVM and ELM) and optimal wavelength selection methods (PCA loadings and SPA) to identify and visualize CRISPR/Cas9-induced mutant rice seeds. PCA was first conducted to find identify clusters between WT and mutants. The score images acquired from PCA on the hyperspectral data reveal accredited discriminations between WT and mutants from two types of rice varieties. Moreover, a PCA loadings method and SPA were applied to extract effective features that were valuable for discrimination. After the appropriate data pretreatment, the SPA-SVM model had robust and valuable calibration and predication capacities, with a classification accuracy of approximately 90%. Finally, the classification of mutant seeds could be visualized on prediction maps by predicting the features of each pixel on individual hyperspectral images. The use of NIR hyperspectral imaging combined with chemometrics and image processing technology for screening CRISPR/Cas9-induced mutants is a very attractive platform and has the potential to be widely used in rapid plant breeding programmes for screening, as it is non-invasive and cost-effective and its does not require any pretreatments.

## Materials and Methods

### CRISPR/Cas9-induced rice samples

**Figure 4:**
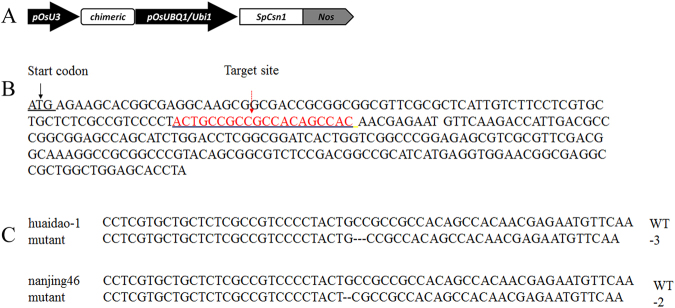
Using CRISPR/Cas 9 to edit the *TWG*6 gene. (**A**) The schematic diagram of the vector used in this study. pOsU3: U3 promoter; chimeric: gRNA + target; pOsUBQ1/Ubi1: UBI promoter; SpCsn1: Cas9 protein; Nos: Nos terminater. (**B**) The sequence of the *TGW6*, the sequence of target site has been labeled by the red colour. (**C**) DNA PCR analysis and sequencing results from huaidao-1 and nanjing46 rice wild-type and mutants.There are 3 bp and 2 bp missing in *TGW6* gene-edited mutants of huaidao-1 and nanjing46, respectively.

The gene-edited mutant rice seeds and their acceptor lines were provided by Zhejiang Academy of Agriculture Science, China. Rice varieties huaidao-1 and nanjing46 were used as the genetic transformation receptors. We designed a vector to target the gene *THOUSAND-GRAIN WEIGHT 6* (*TGW6*)^19^ for both huaidao-1 and nanjing46. *TGW6*, the major QTL for thousand-grain weight, encodes a protein with indole-3-acetic acid (IAA)-glucose hydrolase activity. Loss of function of the *TGW6* allele enhances grain weight through pleiotropic effects on source organs and leads to significant yield increases^19^. The gRNA target sequence was inserted into the Cas9/gRNA plasmid. The constructed plasmid and target sites are shown in Fig. 4(A,B). There were no other differences in the seeds between their transformation receptors and the corresponding mutants.

To detect the effect of the gene-editing, the genome DNA was extracted from each edited plant using the SDS method, and the target gene was sequenced. Each PCR mixture (30 μl volume) consisted of 10× PCR buffer (Takara Biotechnology Co.), 200 mM dNTP (Takara Biotechnology Co.), 0.5 mM of each primer, 1.25 U Taq DNA polymerase (Takara Biotechnology Co.), and template. The PCR programme consisted of a 95 °C/3 min denaturation, followed by 35 cycles of 94 °C/30s, 55 °C/30s and 72 °C/30s, with a final elongation step of 72 °C/7 min. The resulting amplicons were separated by electrophoresis through 2% agarose gels in 0.5× TBE with GelRed staining. As Fig. 4C showed that there were 2 bp and 3 bp missing in the *TGW6* gene-edited mutants of huaidao-1 and nanjing46, respectively.

### Near-infrared hyperspectral image acquisition and calibration

Rice seeds were placed on a controlled conveyer belt and scanned using the NIR hyperspectral imaging system (ImSpector N17E; Spectral Imaging Ltd., Oulu, Finland). The devices of this instrument include a high-performance CCD camera (Hamamatsu, Hamamatsu City, Japan), an imaging spectrograph (ImSpector N17E; Spectral Imaging Ltd., Oulu, Finland) with a wavelength range from 874.41 to 1733.9100 nm, two 150 W tungsten halogen lamps (Fiber-Lite DC950 Illuminator; Dolan Jenner Industries Inc., Boxborough, MA, USA) for illumination, a moving platform controlled by a stepper motor (Isuzu Optics Corp., Taiwan, China) for sample motion, and a computer.

Before acquiring nondeformable and clear NIR images, the exposure time of camera, the distance between the CCD camera and conveyor, and the speed of the conveyor moment were adjusted to 3.2 ms, 28.7 cm and 23 mm/s, respectively. NIR hyperspectral images form a three-dimensional structure (x, y, λ) of multivariate data for processing and analysis, where x and y are the spatial dimensions (numbers of rows and columns in pixels), and λ represents the number of wavebands. Hyperspectral images of the rice seeds had a spectral resolution of 5 nm and 256 spectral channels.

**Figure 5:**
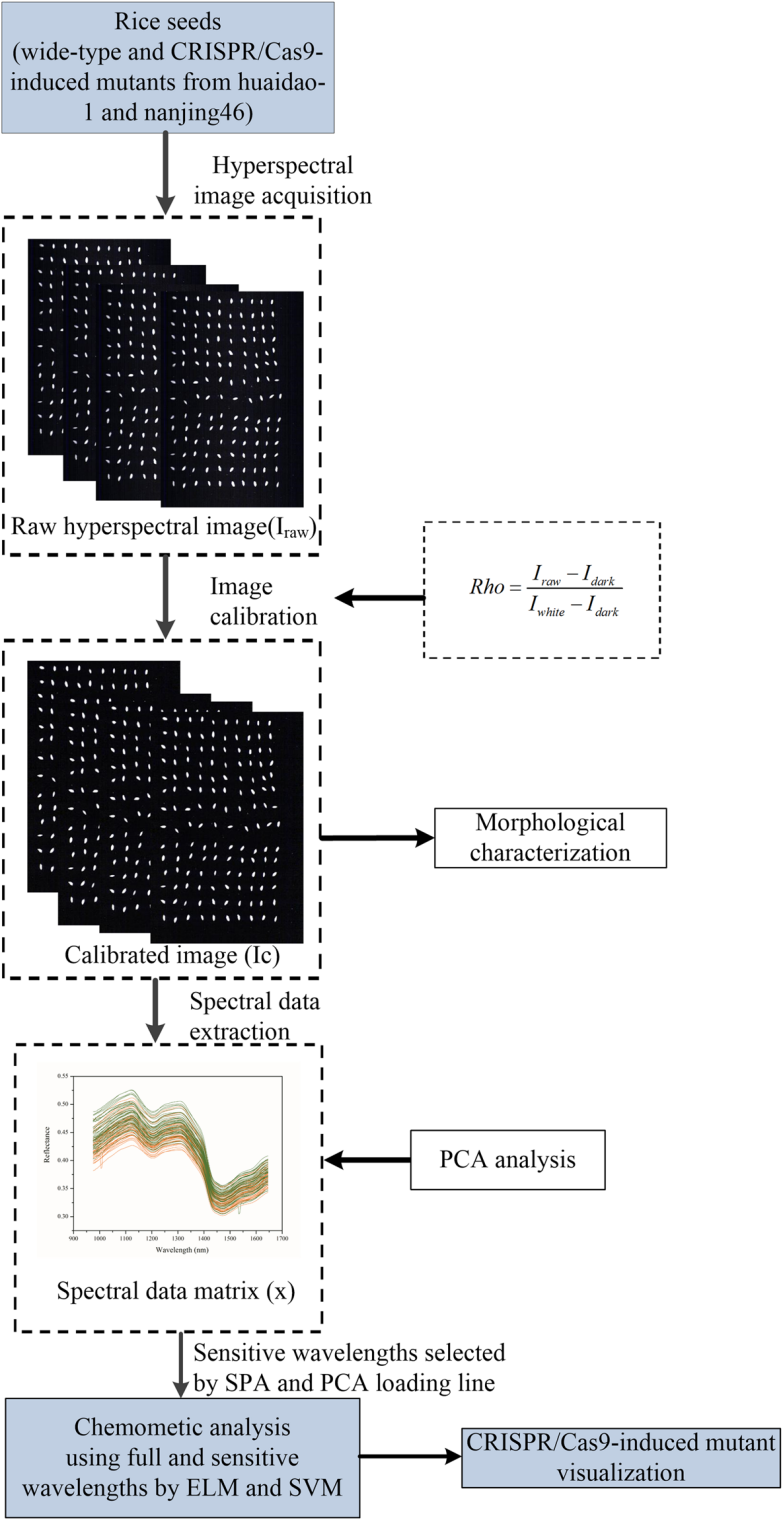
Flowchart of image processing and data analysis for discrimination of CRISPR/Cas9-induced mutants of rice.

For correcting the effects of light source and obtaining data reflection percentage (Rho), raw hyperspectral images (I_raw_) were calibrated with two reference standards using the following equation:

1 $$Rho=\frac{{I}_{raw}-{I}_{dark}}{{I}_{white}-{I}_{dark}}$$

where a dark reference image (I_dark_) was acquired by turning off the light source together with covering the camera lens completely with its opaque cap to remove the influence of dark current in the camera. The white reference image (I_white_) was acquired using a standard white Teflon tile with nearly 100% reflectance. All of the corrected hyperspectral images were then used for spectral information extraction, principal component images acquisition, discrimination purposes, and image visualization. The key steps for discrimination of the CRISPR/Cas9-induced mutants and extracting the morphological characterization of rice are presented in Fig. 5.

### Morphological characterization and thousand-grain weight analysis

The *TGW6* gene is believed to change rice seed shape and organ size^32^, therefore, the morphological characterization from the sample was extracted from the hyperspectral image using an image processing technique. A total of four features (area, perimeter, length, width) from 130 seeds of each rice varieties were extracted. The hyperspectral image at a wavelength of 1139.26 nm was transformed to a greyscale image for later morphological characterization. The four morphological characterizations are as follows:

Perimeter: the number of contour pixels for each seed;

Area: the total number of pixels inside the seed contour pixels;

Length: the number of pixels that specifies the major axis length of the seed;

Width: the number of pixels that specifies the minor axis length of the seed.

Five independent samples of 100 seeds were measured, and the means were converted to thousand-grain weight (TGW).

### Spectral data extraction and pretreatment

Before spectral data extraction, the whole rice seed was segmented from the background and defined as the region of interest (ROI). The reflectance of the rice samples in the ROI was averaged and was taken as the mean spectrum of the relative sample. Spectra were obtained from all hyperspectral images of seeds and were saved in a spectral matrix (*X*). To improve the signal-to-noise ratio and the distinguishing capacity of the models, raw spectra were subjected to noise suppression by wavelet transformation^33^ using Daubechies 8 with decomposition scale 3.

A total of 660 intact samples of each variety were selected for image acquisition. The dataset of each variety was divided into a calibration set and a predication set at a ratio of 2:1 using the Kennard-Stone algorithm^34^. Thus, there were 440 seeds used as the calibration set, and the remaining 220 seeds were used as the prediction set for each variety. All rice seeds were given a category assignment; the wild-type seeds were assigned 1, and the mutants were assigned 2.

### Chemometrics and data analysis

Because there is a large amount of hyperspectral image data containing hidden information, a reliable method is needed to process and extract the features of the spectra. The first step involving analysis was performed using an exploratory principal components analysis (PCA)^35^. The PCA was applied to reduce the spectral dimensions of the hyperspectral image data by projecting the data into a low dimensional subspace, and each of the spectrums was projected in an alternative set of coordinates called the principal components (PCs). The scores of the most significant PCs corresponding to each NIR spectrum were used. The number of PCs was less than or equal to the number of original variables. From the PCA score plot, it is possible to observe the clusters and trends from the different samples.

Variable (wavelength) selection was performed using wavelength selection-based techniques to facilitate and speed up the classification in the next stage of this study. The spectral bands in hyperspectral images are highly correlated; thus, contiguous spectral bands may contain redundant information. Therefore, it is advantageous to extract feature components to perform better predictions and a simpler process. However, inspection of characteristic spectra allows the analysis of similarities and differences between WT and mutants. The PCA loading lines method and a successive projections algorithm (SPA)^36^ were applied to select optimal wavelengths. Loading values resulting from the PCA of the raw spectral data represent the regression coefficient and indicate the most dominant wavelength. Therefore, peaks and valleys based on the wavelength-loading map were selected as important wavelengths. SPA is a forward variable selection algorithm that is designed to select optimal wavelengths with minimal redundancy to address collinearity problems. The optimal wavelengths are finally determined according to the minimum root mean square error of validation (RMSEV) in the validation set of an MLR calibration. The SPA was described in detail in the literature^37^.

In successive stages, support vector machine (SVM) and extreme learning machine (ELM) discrimination models were applied on the raw spectral data, and optimal wavelengths were then selected to screen and distinguish the WT and mutant strains. SVM is a supervised learning model that has been widely used for classification^38^. Compared with other machine learning methods, this method can develop models with fewer training samples and can overcome the local minimum in a neural network. Detailed information on this popular model can be found in the literature^38^. An SVM model with the radial basis function (RBF) as a kernel function was used in this case, and different penalty parameters (c) and kernel function parameters (g) were chosen to achieve the highest recognition rate. ELM, a feedforward neural network, can also be applied in binary classification applications^39^. ELM operates with a single layer of hidden nodes, where the weights connecting inputs to hidden nodes are randomly assigned and never updated. In this paper, optimal numbers of hidden nodes for training were determined from 1 to 80 in steps of 1 to have the best discrimination capability. The performances of the discrimination models were evaluated based on the classification accuracies of the calibration set and the prediction set.

### CRISPR/Cas9-induced mutant visualization

Finally, a very uniform set of seeds from different genotype were presented to the optimal classification model and tested in order to inspect the robustness of the model. To acquire these validation data, the exposure time of camera, the distance between the CCD camera and conveyor, and the speed of the conveyor moment of the NIR hyperspectral imaging system were adjusted to 4 ms, 15.1 cm and 16 mm/s, respectively. The major advantage of hyperspectral imaging is to provide spatial and spectral information simultaneously, which facilitates visualizing the distribution of the features in the tested sample. In the current study, an optimal classification model combined with hyperspectral image processing was used to map and visualize each pixel of the hyperspectral images to demonstrate mutants. This visualization model was run by calculating the dot product between the spectrum of each pixel in the image and the accuracy rate obtained from the optimal model base on particular wavelengths. The classification prediction map is displayed in colours, where each colour represents the corresponding rice varieties.

### Software tools

NIR hyperspectral images were analysed using the ENVI version 4.6 Hyperspectral image analysis soft package (ITT, Visual Information Solutions, Boulder, CO, USA). Shape features extraction, NIR spectral extraction, multivariate data analysis and all stages involved in image visualization purposes were applied using MATLAB version R2010b (The Math-Works, Natick, MA, USA). Statistical comparisons were made using a one-way analysis of variance (ANOVA). The differences between means were established using the Student-Newman-Keuls tests (p < 0.05) in the SPSS version 18.0 statistical programme (SPSS Inc., Chicago, IL, USA). In addition, graphs were prepared using origin Pro 8.5 SR0 (Origin Lab Corporation, Northampton, MA, USA) software.
